# Editorial: Computational approaches for non-coding RNA prediction studies

**DOI:** 10.3389/fmolb.2022.1091918

**Published:** 2022-12-13

**Authors:** Jia Qu, Xiao-Long Cheng

**Affiliations:** School of Computer Science and Artificial Intelligence, Aliyun School of Big Data, School of Software, Changzhou University, Changzhou, China

**Keywords:** machine learning, complex network, non-coding RNA, prediction, computational model

## Introduction

It is well established that non-coding RNA (ncRNAs) may affect normal gene expression and disease progression, and become a new class of targets for drug discovery (Anastasiadou et al., 2018). Different classes of ncRNAs with various activities are of great importance for cellular function and development. In particular, the deficiencies or excesses of ncRNA have been associated with a variety of complex human diseases, including myocardial infarction, virus infection, Alzheimer’s disease, metabolic diseases, cancers, and many others (Slack and Chinnaiyan, 2019). Changes in the expression of ncRNAs during treatment could be used as biomarkers for therapeutic response (Mercer et al., 2009; Ning et al., 2021). Therefore, it is necessary to identify disease-related ncRNAs. Moreover, an increasing number of researchers are studying the regulatory interactions between ncRNAs classes, as well as the associations between ncRNA and other biological entities. Certainly, predicting ncRNA related networks will greatly expand our understanding of ncRNA functions and their coordinated regulatory networks (Chen et al., 2019).

Traditional methods for ncRNA prediction are not only time-consuming but also random and blind (Chen et al., 2020). With the increasing popularity of artificial intelligence (AI) in ncRNA prediction and the massive increase of ncRNA related multi-source biological data, various computing models have been proposed for the prediction of ncRNA. An essential outcome of this development is that our work in ncRNA prediction studies has considerably accelerated.

The following articles in this Research Topic address the problem of prediction of ncRNA based on computational methods.

In the article entitled “*5-Methylcytosine RNA Methyltransferases-Related Long Non-coding RNA to Develop and Validate Biochemical Recurrence Signature in Prostate Cancer*” Wang et al. the authors identified and characterized a series of m5C-related long non-coding RNAs (lncRNAs) in prostate cancer (PCa). Univariate Cox regression analysis and least absolute shrinkage and selector operation (LASSO) regression analysis were implemented to construct a m5C-related lncRNA prognostic signature. The study revealed that the m5C-related lncRNA signatures could precisely predict biochemical recurrence in patients with PCa by the score of the proposed model of m5c-lnc.

In the article entitled “*Ranolazine Inhibits Pyroptosis via Regulation of miR-135b in the Treatment of Diabetic Cardiac Fibrosis*” Ren et al. the authors elucidated the mechanism of action of ranolazine against diabetic cardiac fibrosis and to investigate the role of miR-135b in this process. They reported for the first time that hyperglycemia decreases miR-135b expression in cardiac fibroblasts, and ranolazine treatment of diabetic cardiac fibrosis is mediated by the miR-135b/caspase-1/TGF-β1 pathway. The study provided a solid theoretical basis for understanding the pathogenesis of diabetic cardiac fibrosis and the clinical use of ranolazine in the treatment of diabetic cardiomyopathy.

The article entitled “*Inferring microRNA regulation: A proteome perspective*” Ofer and Linial presented a different paradigm for predicting miRNA-regulated genes based on the encoded proteins by using a supervised machine-learning approach. The model highlighted the value of information embedded in the functional proteome in revealing the complexity of regulation by microRNA (miRNAs). The article concluded that protein function is informative across species in predicting post-transcriptional miRNA regulation in living cells. In the future work, a similar AI-based approach will be useful for creating a generalized model for post transcriptional regulation in living cells by ncRNAs (e.g., lncRNA, circRNA).

The article entitled “*Characterizing the molecular heterogeneity of clear cell renal cell carcinoma subgroups classified by miRNA expression profile*” Shen et al. performed unsupervised clustering using TCGA-retrieved prognostic miRNAs expression profiles. By assessing principal component analysis (PCA), t-distributed stochastic neighbor embedding (t-SNE), and consensus heatmaps, two clear cell renal cell carcinoma (ccRCC) subtypes were identified. The work described the molecular heterogeneity among ccRCC cancers and provided potential targets used as effective biomarkers for ccRCC diagnosis and prognosis.

## Conclusion

These articles highlighted the important role of ncRNAs in the regulation of gene expression by interactions between ncRNAs and other biological molecules. Computational methods can predict potential outcomes based on multi-source data from experiments. These articles cover the latest advancements in ncRNA research. In particular, ncRNAs include miRNAs, lncRNAs, circRNAs (circular RNAs), snRNAs (small nuclear RNAs), and small interfering RNA (siRNA). Among these, miRNAs and lncRNAs have been the most frequently studied, and the articles in this Research Topic place a special emphasis on miRNAs and lncRNAs. In the future, attention should be given to other ncRNAs and not be limited to miRNAs and lncRNAs.
